# Wild-type and SAMP8 mice show age-dependent changes in distinct stem cell compartments of the interfollicular epidermis

**DOI:** 10.1371/journal.pone.0215908

**Published:** 2019-05-15

**Authors:** Gopakumar Changarathil, Karina Ramirez, Hiroko Isoda, Aiko Sada, Hiromi Yanagisawa

**Affiliations:** 1 Life Science Center for Survival Dynamics, Tsukuba Advanced Research Alliance (TARA), University of Tsukuba, Tsukuba, Japan; 2 Graduate School of Comprehensive Human Sciences, University of Tsukuba, Tsukuba, Japan; 3 Ph.D. Program in Human Biology, School of Integrative and Global Majors, University of Tsukuba, Tsukuba, Japan; 4 Alliance for Research on the Mediterranean and North Africa (ARENA), University of Tsukuba, Tsukuba, Japan; 5 Faculty of Life and Environmental Sciences, University of Tsukuba, Tsukuba, Japan; 6 Faculty of Medicine, University of Tsukuba, Tsukuba, Japan; National Center for Toxicological Research, UNITED STATES

## Abstract

Delayed wound healing and reduced barrier function with an increased risk of cancer are characteristics of aged skin and one possible mechanism is misregulation or dysfunction of epidermal stem cells during aging. Recent studies have identified heterogeneous stem cell populations within the mouse interfollicular epidermis that are defined by territorial distribution and cell division frequency; however, it is unknown whether the individual stem cell populations undergo distinct aging processes. Here we provide comprehensive characterization of age-related changes in the mouse epidermis within the specific territories of slow-cycling and fast-dividing stem cells using old wild-type, senescence-accelerated mouse prone 1 (SAMP1) and SAMP8 mice. During aging, the epidermis exhibits structural changes such as irregular micro-undulations and overall thinning of the tissue. We also find that, in the old epidermis, proliferation is preferentially decreased in the region where fast-dividing stem cells reside whereas the lineage differentiation marker appears to be more affected in the slow-cycling stem cell region. Furthermore, SAMP8, but not SAMP1, exhibits precocious aging similar to that of aged wild-type mice, suggesting a potential use of this model for aging study of the epidermis and its stem cells. Taken together, our study reveals distinct aging processes governing the two epidermal stem cell populations and suggests a potential mechanism in differential responses of compartmentalized stem cells and their niches to aging.

## Introduction

Aging is a gradual decline in physiological functions over a course of time. It still remains a mystery what are the crucial drivers for aging at cellular and molecular levels. Among a myriad of hypotheses proposed, a theory of stem cell aging suggests that aging is caused by the inability of adult stem cells to replenish tissues due to depletion or accumulation of molecular and cellular defects [1–3].

The interfollicular epidermis (IFE) is the uppermost layer of the skin, separated from dermis by the basement membrane. A rapid turnover and repair of the IFE is sustained by heterogeneous populations of stem cells located in the basal layer, which proliferate and differentiate into upper spinous, granular and cornified layers [4]. During aging, the morphological and functional changes become apparent in the mouse and human IFE, including a decrease in epidermal thickness [5], flattening of epidermal-dermal junction [6, 7], impaired barrier function [8] [9] and delayed wound healing [10]. However, it remains unclear how aging affects stem cells residing at the epidermal basal layer and how they contribute to age-related epidermal tissue dysfunction.

Tissue stem cells are largely considered to be slow-cycling in nature, which is proposed to have a protective role on stem cells, by preventing them from replication-induced DNA damage or telomere shortening and ensuring the maintenance of a healthy pool of stem cells that replenish the tissue for a long term [11]. In contrast, we have previously proposed that slow-cycling and fast-dividing cells act as two distinct populations of stem cells within the specific territory of the IFE [12]. These stem cell territories are most evident in the tail IFE, which is composed of alternating and regularly spaced interscale and scale IFE. The interscale IFE harbors slow-cycling stem cells that undergo orthokeratotic differentiation, a typical process of epidermal keratinization. The scale IFE harbors fast-dividing stem cells that go through parakeratotic differentiation characterized by the lack of granular layer and retention of nucleus in the cornified layer [12, 13]. However, it remains largely unknown how aging affects the stem cells with respect to their division frequency and their microenvironment.

It has been controversial whether stem cells in the IFE undergo aging or not. Despite the appearance of age-dependent structural changes in the epidermis, epidermal stem cells are well maintained in the mouse IFE [7]. In vitro studies with mouse keratinocytes indicate that young and old mouse epidermal keratinocytes show similar growth characteristics [14]. Recent studies in mice showed that there are no transcriptional or morphological changes under homeostatic conditions in the aged dorsal skin [10], whereas in the paw skin, hyperproliferation phenotype is observed during aging [15]. In the case of human beings, there are age-dependent changes in growth potential, the number and kinetics of transit-amplifying cells [16], expression of genes associated with stem cells [17] or cellular senescence [18–20]. Nevertheless, previous studies haven’t addressed these changes in heterogeneous stem cell populations and their niches.

Genetic approaches have identified genes and factors that are responsible for some aspects of the IFE aging [21–24] [15, 25–27]; however, single gene mutation may not fully explain the aging process because of the complex and multifactorial nature of aging.

A senescence-accelerated mouse (SAM) was generated by selective inbreeding of AKR/J strain, resulting in the establishment of 9 strains of senescence-prone (SAMP) mice and 3 senescence-resistant (SAMR) mice with strain-unique phenotypes of accelerated aging [28]. In the skin, SAMP1 strain is reported to exhibit an increase in elastic fibers and epidermal thickness at 1 to 1.5 year of age [29], which resemble characteristics of human photoaging, a skin damage resulting from prolonged sunlight exposure [30, 31]. SAMP8 strain shows neurodegenerative and cognitive deficits associated with Alzheimer’s disease, and abnormality in circadian rhythms along with a shorter lifespan [32–34]. A recent study has reported that SAMP8 mice, when fed with a carbohydrate-restricted diet, showed thinning of skin with an increase in senescence indicators and the mTOR activity [35]. Nevertheless, the existing reports regarding SAMP8 mice remain elusive regarding the age-related changes in the epidermis and its stem cells.

In this study, we characterized age-dependent changes of slow-cycling and fast-dividing epidermal stem cells using the tail epidermis of young and old C57BL/6 strain of wild-type mice. Moreover, in the aim of finding a new system to study epidermal stem cell aging, we utilized SAMP1 and SAMP8 strains of senescence-prone mice and compared their phenotypes with the process of natural aging in wild-type mice.

## Materials and methods

### Mice

All mouse experiments were performed according to the protocols approved by Animal Care and Use Committee in the University of Tsukuba. C57BL/6 mice, both young (2-month-old) and old (2-year-old) were purchased from Charles River Laboratories or Japan SLC, Inc. The mice were housed in Laboratory Animal Resource Center, University of Tsukuba, prior to experiments. The AKR/J strain of senescence-resistant SAMR1/TaSlc and senescence-prone SAMP1/SkuSlc and SAMP8/TaSlc mice were obtained from Japan SLC, Inc. SAMR1, SAMP1, and SAMP8 mice were purchased at 6 months of age and were immediately sacrificed or housed in Laboratory Animal Resource Center, University of Tsukuba until they reached 1-year-old. Both male and female mice were used for experiments.

### BrdU treatment

For the labeling of proliferative cells, BrdU (5-Bromo-2’-deoxyuridine; Sigma-Aldrich, B5002) was administered ad libitum in drinking water (0.8 mg/ml) for 2 days prior to sacrifice.

### Hematoxylin and eosin (H&E) staining

For histological assessment, the tail skin was directly embedded in optimal cutting temperature (O.C.T.) compound (Tissue-Tek, Sakura). Ten-micron sections were fixed in 4% Paraformaldehyde (PFA) at room temperature for 10 minutes. Subsequently, the sections were washed and stained with hematoxylin (Wako, 131–09665) for 20 minutes and eosin Y (Wako, 058–00062) for 15 seconds, dehydrated and mounted in Entellan new mounting solution (Merck Millipore, HX73846161). Microscopic observation was performed using a Zeiss Axio imager Z2 and tiled images of tail sections were acquired with Zen 2.3 Pro software.

### Immunostaining of skin sections

The tail skin sections were subjected to immunostaining as previously reported [12]. Primary antibodies were used at the following dilutions: rabbit anti-K14 (1:1000, BioLegend, 905304), goat anti-PDGFRα (1:100, R&D systems, AF1062), rat anti-α6-integrin (1:150, BD biosciences, 555734), rabbit anti-Collagen IV (1:100, Chemicon, AB756P), rat anti-BrdU (1:300, Abcam, ab6326), mouse anti-K10 (1:500, Abcam, ab9026) and guinea pig anti-K31 (1:100, PROGEN Biotechnik, GP-hHa1). To stain with anti-BrdU antibody, skin sections were incubated with 1N HCl at 37°C for 1 hour after blocking. Preparations were analyzed and imaged using a fluorescent microscope (Zeiss, Axio Imager.Z2). The brightness and contrast of images were adjusted with equal intensity among different mouse groups by using Adobe Photoshop.

### Whole-mount immunostaining of the mouse tail epidermis

Tail whole-mount tissues were processed as previously described [12]. Primary antibodies were used at the following dilutions: rat anti-β4-integrin (CD104) (1:100, BD Biosciences, 553745), rabbit anti-caspase-3, active (cleaved) form (1:100, Millipore, AB3623), rat anti-BrdU (1:300, Abcam, ab6326), rat anti-Ki67 (1:100, eBioscience, 14-5698-82), rabbit anti-K14 (1:1000, BioLegend, 905304, polyclonal), mouse anti-K10 (1:100, BioLegend, 904301, or 1:100, Abcam, ab9026), guinea pig anti-K31 (1:100, PROGEN Biotechnik, GP-hHa1). All secondary antibodies (Alexa 488 or Alexa 546, Invitrogen) were used at 1:200 dilution. The mouse primary antibodies were blocked with MOM kit (Vector Laboratories). All samples were counterstained with Hoechst (Sigma, B2261) for 1 hour and mounted. To stain with anti-BrdU antibody, tail whole-mount pieces were blocked and incubated with 2N HCl at 37°C for 1 hour, further washed and stained. The stained whole-mount epidermis was observed by a confocal microscope (Zeiss LSM 700) with a ZEN 2010 software. All pictures were shown as projected Z-stack image, viewed from the basal side.

### Quantification, statistical analysis and reproducibility

All experiments with or without quantification were independently performed at least three times with different mice and the representative data are shown. All quantifications were independently performed on ≥3 mice. Data are shown as means ± standard error of the mean (S.E.M.). To minimize the possible effects of hair cycle differences to IFE [36], anagen and catagen skin were excluded from quantification analyses.

**Fig 1:**
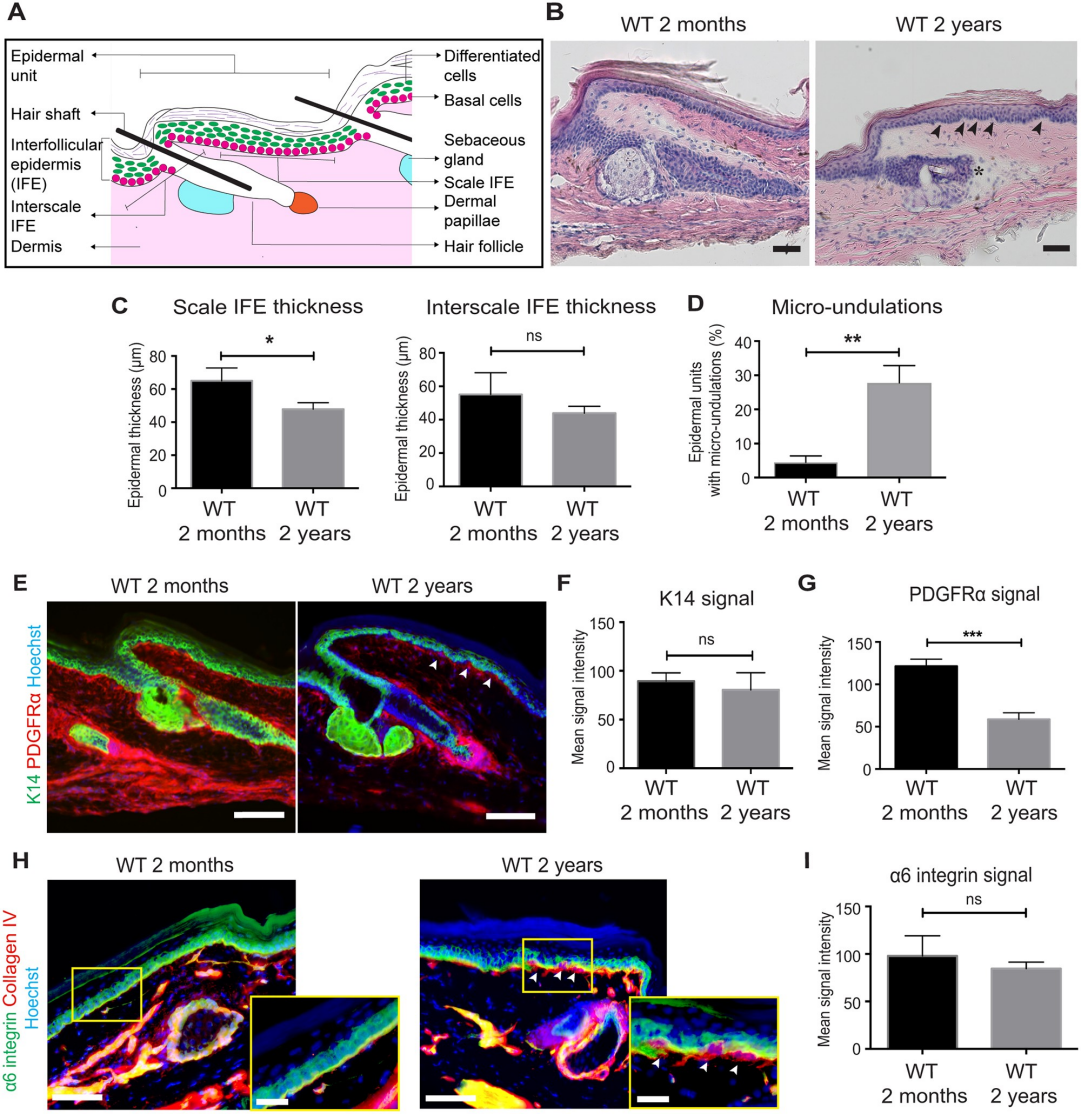
Age-associated structural changes in the tail epidermis. (**A**) Schematic diagram of the mouse tail skin. (**B**) Hematoxylin-eosin stained tail skin sections of young (2-month-old) and old (2-year-old) mice. Arrowheads indicate the micro-undulation in the basal region. Degenerating hair follicle is observed (asterisk). Scale bars: 50 μm. (**C**) Quantification of the thickness of the scale and interscale IFE. The epidermal thickness is quantified from ≥6 epidermal units per mouse. *N* = 3. Error bars show S.E.M. Student’s t-test. *; *P* < 0.05, ns: not significant; *P* = 0.22 (C, the interscale IFE). (**D**) The proportion of epidermal units harboring micro-undulations. ≥30 epidermal units are counted in each mouse to score micro-undulations of basal regions of the IFE. *N* = 3. Student’s t-test is used. **; *P* < 0.01. (**E**) Immunostaining of tail skin sections of young (2-month-old) and old (2-year-old) mice with K14 (basal cell marker, green) and PDGFRα (dermal cell marker, red) antibodies. Hoechst is used to stain nucleus (blue). Arrowheads indicate the micro-undulation. Scale bars: 100 μm. (**F, G**) Quantification of the mean signal intensity of K14 (F) or PDGFRα (G). The intensity of immunostaining signals in skin section is measured and averaged from ≥50 individual cells per mouse. *N* = 3. Student’s t-test. ***; *P* < 0.001. ns: not significant; *P* = 0.46 (F). (**H**) Immunostaining of tail skin sections of young (2-month-old) and old (2-year-old) mice with α6 integrin (basal cell marker, green) and Collagen IV (basement membrane marker, red). Scale bars: 100 μm. The area within the yellow box is shown with higher magnification. Scale bars: 20 μm. (**I**) Quantification of the mean signal intensity of α6 integrin. The intensity of immunostaining signals in skin section is measured and averaged from ≥50 individual cells per mouse. *N* = 3. Student’s t-test. ns: not significant; *P* = 0.35 (I).

In H&E-stained tail sections, an epidermal unit was defined as the IFE region between adjacent hair follicle structures (Fig 1A) and ≥30 epidermal units were manually counted in each mouse to score micro-undulation of basal regions of the IFE. The epidermal thickness was quantified from ≥6 epidermal units per mouse using Zen 2.3 Pro software. The measurements for scale IFE thickness were made at the center of an epidermal unit, and interscale IFE thickness were made on the interfollicular epidermal region adjacent to the hair follicles (Fig 1A). The intensity of immunostaining signals in skin section was measured and averaged from ≥50 individual cells using ImageJ software. The caspase3+ basal cells were manually scored from ≥50 interscale or scale IFE structures per mouse. The number of BrdU+ cells were manually scored from ≥8 interscale or scale IFE structures in projected Z-stack images or ≥100 basal cells on skin sections.

Student’s two-tailed t-tests or One-way ANOVA were performed to compare wild-type young vs old, or SAMR1, SAMP1 and SAMP8, respectively, by using GraphPad Prism6 software. For the quantification of caspase3+ basal cells, Mann-Whitney U test (wild-type young and old), and while Kruskal-Wallis test for multiple comparison (SAMR1, SAMP1 and SAMP8) are used. A significant difference is defined as *P* < 0.05.

### Flow cytometry

Mouse tail skin was incubated in 0.25% Trypsin/EDTA overnight at 4°C and for 30 min at 37°C. Single cell solution was prepared by scraping and subsequent filtering with strainers (70 μm, followed by 40μm). Cells were stained with the following antibodies for 30 min on ice: CD34-biotin (1:50, eBioscience, 13–0341), Streptavidin-APC (1:100, BD Biosciences, 554067), α6-integrin-BUV395 (1:100, BD Biosciences, custom order) and Sca1-BV421 (1:100, BD Biosciences, 562729). The dead cells were excluded by using propidium iodide (P4864, Sigma Aldrich). FACS (Fluorescence-activated cell sorting) analyses were performed with FACS Aria (BD Biosciences). The data was processed with FlowJo software to measure the mean fluorescence intensity of basal markers. For the detection of apoptotic cells, cells were stained with PE Annexin V Apoptosis Detection Kit with 7-AAD (BioLegend) according to the manufacturer’s instructions.

## Results

### The irregular micro-undulations and a decreased thickness of the old epidermis

To characterize age-dependent changes in the skin, we chose the tail IFE where stem cell territories were most well-defined (Fig 1A). The C57BL/6 mice at young (2-month-old) and old (2-year-old) age groups were used, which were equivalent to roughly 20–30 years and ~70 years in humans, respectively [37, 38]. Hematoxylin-eosin (H&E) staining showed that the thickness of the IFE was decreased in old mice compared to young mice especially in the scale region (Fig 1B and 1C). In addition, the basal region of the old epidermis was irregular and showed a micro-undulating pattern, hence appearing “wavy” (Fig 1B, arrowheads and Fig 1D). To further characterize the age-associated changes in the IFE, we stained tissues with Keratin (K) 14, α6-integrin (markers of the epidermal basal layer), Collagen IV (a marker of basement membrane) and PDGFRα (a marker of dermal fibroblasts). The staining of K14 and α6-integrin confirmed the histological changes of the IFE (Fig 1E and 1H), but the intensity of basal markers remained unchanged (Fig 1F and 1I). In contrast, the expression of PDGFRα was significantly decreased in the old dermis (Fig 1E and 1G), indicating that dermal fibroblasts were affected by aging as previously reported [39–41]. The basement membrane, visualized by collagen IV staining, appeared thicker in the old IFE where micro-undurations were evident (arrowheads in Fig 1H). Thus, our observation indicates that even though the expression level of basal markers is unaffected during aging, the basal layer of the IFE seems to have gained an undulating wavy pattern, which may be due to alterations in cellular properties of epidermal stem cells located in the basal region.

### Senescence prone SAMP8, but not SAMP1, shows age-associated structural changes in the epidermis

**Fig 2:**
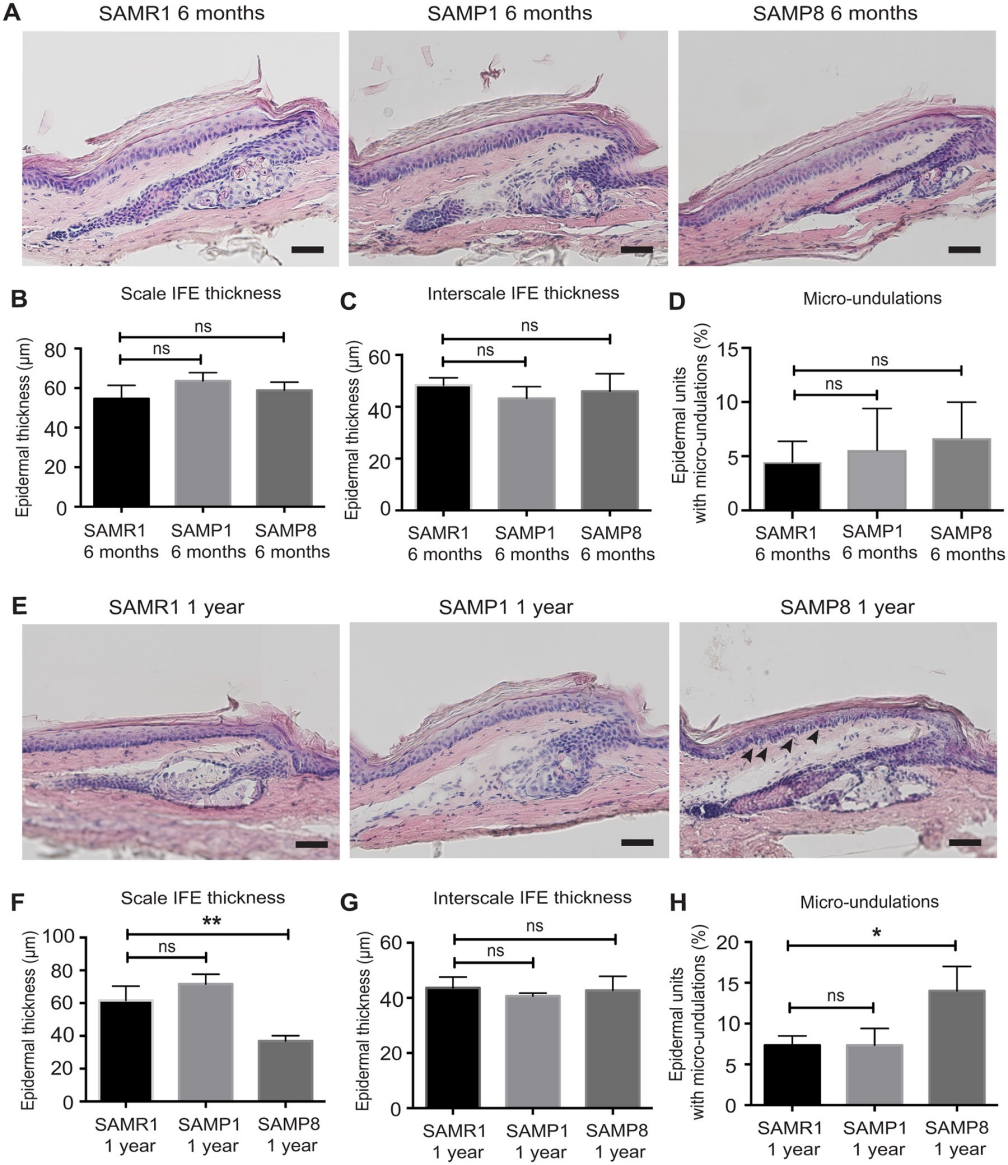
Epidermal structural changes in the SAM mouse model. (**A**) Hematoxylin-eosin stained tail skin sections of senescence-resistant SAMR1 or senescence-prone SAMP1 and SAMP8 strains at 6 months of age. Scale bars: 50 μm. (**B, C**) Quantification of the epidermal thickness. IFE, interfollicular epidermis. The epidermal thickness is quantified from ≥6 epidermal units per mouse. *N* = 3. One-way ANOVA. Error bars show S.E.M. ns: not significant. (B) SAMR1 vs. SAMP1; *P* = 0.13. SAMR1 vs. SAMP8; *P* = 0.53. (C) SAMR1 vs. SAMP1; *P* = 0.40. SAMR1 vs. SAMP8; *P* = 0.79. (**D**) The proportion of epidermal unit harboring the micro-undulation. ≥30 epidermal units are counted in each mouse to score micro-undulations of basal regions of the IFE. *N* = 3. One-way ANOVA. ns: not significant. SAMR1 vs. SAMP1; *P* = 0.87. SAMR1 vs. SAMP8; *P* = 0.61. (**E**) Hematoxylin-eosin stained tail skin sections of senescence-resistant SAMR1, senescence-prone SAMP1 and SAMP8 stains at 1 year of age. Arrowheads indicate the micro-undulations in the basal region. Scale bars: 50 μm. (**F, G**) Quantification of the IFE thickness. The epidermal thickness is quantified from ≥6 epidermal units per mouse. *N* = 3. One-way ANOVA. ns: not significant. (F) SAMR1 vs. SAMP1; *P* = 0.17. SAMR1 vs. SAMP8; **; *P* < 0.01. (G) SAMR1 vs. SAMP1; *P* = 0.56. SAMR1 vs. SAMP8; *P* = 0.94. (**H**) The proportion of epidermal unit harboring the micro-undulation. ≥30 epidermal units are counted in each mouse to score micro-undulations of basal regions of the IFE. *N* = 3. One-way ANOVA. ns: not significant. SAMR1 vs. SAMP1; *P >* 0.99. SAMR1 vs. SAMP8; *; *P* < 0.05.

Next, we addressed whether SAM mice, a model of premature aging, showed accelerated aging phenotypes in the skin IFE. We analyzed the skin phenotypes of senescence prone SAMP1 and SAMP8 mice, which show the average life span of ~9.7 months [28], and compared with senescence resistant SAMR1 mice as controls. To study the onset and time course of possible age-associated phenotypes in the SAM model, 6 months and 1 year of age were used for analyses. The tail skin of SAMP1, SAMP8 and age-matched SAMR1 controls were subjected to H&E staining and immunostaining with α6-integrin and Collagen IV antibodies. At 6 months of age, no apparent changes were observed in the skin at the histological level (Fig 2A–2D). At 1 year of age, SAMP8, but not SAMR1 or SAMP1 mice showed precocious aging phenotypes in the skin IFE: the scale IFE became thinner and the micro-undulations in the basal region were more pronounced (Fig 2E–2H). Similar to the old wild-type IFE (Fig 1H), the thickness of Collagen IV+ basement membrane was found to be increased under the micro-undulations of the SAMP8 strain at 1 year of age, whereas the intensity of the basal marker α6-integrin remained largely unchanged (S1 Fig). Thus, the age-associated changes we observed in the 2-year-old wild-type mice were appeared in SAMP8 mice by 1 year of age, but not in SAMP1 mice.

### Apoptosis of epidermal basal cells remains unchanged with age

**Fig 3:**
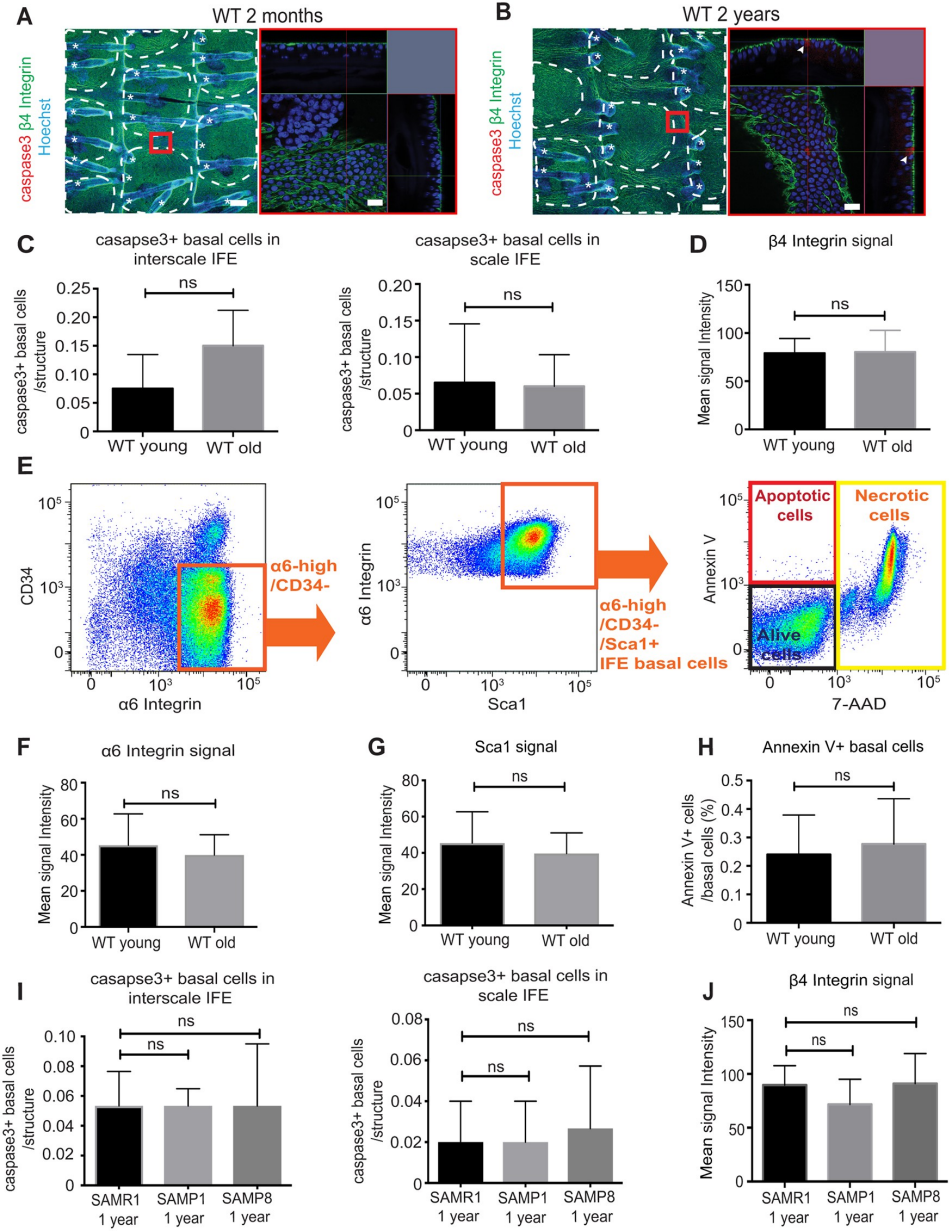
The apoptotosis in the basal layer remains largely unchanged with age. (**A, B**) Tail whole-mount epidermal sheets are stained with cleaved caspase-3 (apoptosis marker, red), β4 integrin (basal cell marker, green) and Hoechst (blue). (A) and (B) represent the immunostaining of wild-type young (2-month-old) and old (2-year-old) mice. The area within the red box is shown with higher magnification. Arrowhead represents apoptotic cells in the basal layer (B). Asterisks represent hair follicles. White dotted lines represent the boundary of scale and interscale. Scale bars: 100 μm (left) or 20 μm (right). (**C**) Quantification of the number of caspase3+/β4 integrin+ cells per an interscale or scale structure. The caspase3+ basal cells are scored from ≥50 interscale or scale IFE structures per mouse. *N* = 4. Error bars show S.E.M. Mann-Whitney U test. ns: non-significant; *P* = 0.22 in interscale IFE; *P* = 0.85 in scale IFE. (**D**) Quantification of the mean signal intensity of β4 integrin. Signal from whole-mount staining shown in (A, B) is measured. *N* = 4. Student t-test. ns: not significant. *P* = 0.93. (**E**) FACS dot plot shows gates to define basal cells in the interfollicular epidermis (α6 integrin+/CD34−/Sca1+), followed by the analysis for 7-AAD and Annexin V signals. (**F, G**) The mean signal intensity of α6 integrin (F) and Sca1 (G) measured by FACS. *N* = 3. Student’s t-test. ns: not significant; *P* = 0.65 (F); *P* = 0.66 (G). (**H**) Graph shows the percentage of apoptotic cells (Annexin V+/7-AAD−) in the basal IFE. *N* = 3. Student’s t-test. ns: not significant; *P* = 0.77. (**I**) Quantification of the number of caspase3+/β4 integrin+ cells in SAM mice at 1-year-old. The caspase3+ basal cells are scored from ≥50 interscale or scale IFE structures per mouse. *N* = 3. Kruskal-Wallis test for multiple comparison is used. ns: non-significant; SAMR1 vs. SAMP1; *P >* 0.99. SAMR1 vs. SAMP8; *P >* 0.99 in the interscale IFE and SAMR1 vs. SAMP1; *P >* 0.99. SAMR1 vs. SAMP8; *P >* 0.99 in the scale IFE. (**J**) Quantification of the mean signal intensity of β4 integrin. Signal from whole-mount staining (S2B Fig) is measured. *N* = 3. One-way ANOVA. ns: not significant; SAMR1 vs. SAMP1; *P* = 0.57. SAMR1 vs. SAMP8; *P* = 0.99.

Apoptosis plays a pivotal role in the maintenance of the fitness of rapidly-renewing epidermis and prevention of skin diseases, such as psoriasis or carcinogenesis [42]. Studies also suggest that there is an increased number of cells harboring DNA damage or apoptotic cells in the basal and spinous layers of the old human epidermis [43, 44]. Therefore, micro-undulated and “wavy” basal region of the old epidermis may be due to the increased apoptosis and subsequent loss of epidermal basal cells. To test this possibility, we performed whole-mount staining of tail epidermal sheets with active caspase-3 and β4 integrin antibodies to label apoptotic cells within the basal layer of the IFE (Fig 3A and 3B). The staining with active caspase-3 antibody occasionally detected cells that were positive for both β4 integrin and caspase-3 in old wild-type mice (arrowhead, Fig 3B), indicating the presence of apoptotic cells in the basal layer. However, these apoptotic cells were rare and no significant differences were detected between wild-type young and old mice (Fig 3C). Consistent with K14 and α6-integrin staining (Fig 1E and 1H), the expression pattern and the signal intensity of β4 integrin were unchanged between young and old mice (Fig 3A, 3B and 3D).

This observation was further supported by the flow cytometry analysis, in which the old IFE showed no significant changes in the number of AnnevinV+ apoptotic cells nor the signal intensity of basal markers, α6-integrin and Sca1 (Fig 3E–3H). Similarly, SAMP1/P8 mice showed no significant increase of apoptotic cell death in the basal IFE (Fig 3I–3J, S2 Fig). Hence, we concluded that apoptosis of basal cells was negligible to cause any functional and structural changes in the mouse IFE during aging.

### Age-dependent decrease in proliferation within fast-dividing stem cell compartment

**Fig 4:**
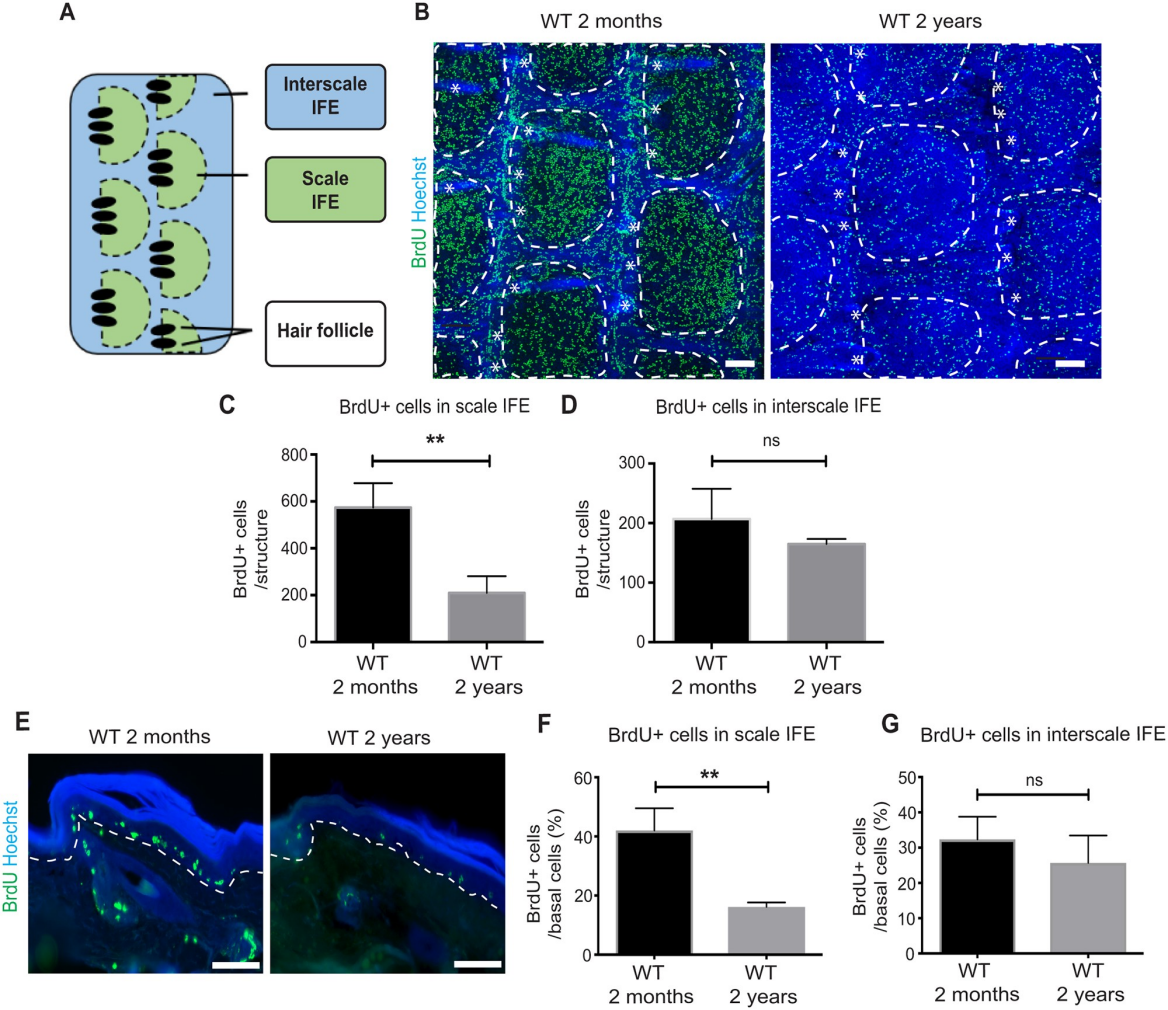
Age-dependent decrease in proliferation in the scale epidermis. (**A**) Schematic view of the interscale and scale structures in the tail whole-mount epidermis. (**B**) Tail whole-mount epidermal sheets are stained with BrdU (proliferation marker, green) and Hoechst (blue). White dotted lines represent the boundary of scale and interscale. Asterisks represent hair follicles. Scale bars: 100 μm. (**C, D**) Quantification of BrdU-positive cells within each interscale and scale structure. The number of BrdU+ cells is scored from ≥8 interscale or scale IFE structures per mouse. *N* = 3. Error bars show S.E.M. Student’s t-test is used. **; *P* < 0.01 (C). ns: not significant; *P* = 0.22 (D). (**E**) BrdU (proliferation marker, green) and Hoechst (blue) staining in the tail skin sections. (**F, G**) Proportion of BrdU+ cells per basal cells in the scale (F) or interscale IFE (G). The number of BrdU+ cells is scored from ≥100 basal cells per mouse. *N* = 3. Student’s t-test. ns: not significant; **; *P* < 0.01 (F), and *P* = 0.31 (G).

The old human skin shows a decrease in the basal cell density along with the decreased proliferation of epidermal basal cells [17]. It has also been reported that proliferation is decreased in the tail IFE of old mice [7]. To understand the age-dependent proliferation changes with regard to stem cell compartments in the tail IFE, we performed BrdU labeling of the epidermis. Wild-type young and old mice were treated with BrdU for 2 days to ensure the enrichment of BrdU+ cells in the scale IFE, which corresponded to the localization of fast-dividing epidermal stem cells (Fig 4A) [12]. In the whole-mounts of old wild-type mice, the number of BrdU+ cells appeared sparse in the tail IFE (Fig 4B). Interestingly, we found a significant decrease in BrdU+ cells in the scale IFE, a location of fast-dividing epidermal stem cells (Fig 4B and 4C). In contrast, the aged interscale IFE did not show a substantial decrease in BrdU+ cells in comparison to the young interscale IFE (Fig 4B and 4D). Similarly, the section staining showed that the proportion of BrdU+ basal cells was significantly decreased in the scale IFE, but not in the interscale IFE (Fig 4E–4G). These results indicate that with age, proliferation of fast-dividing epidermal stem cells is more profoundly affected compared to the slow-cycling stem cells, probably due to their frequent cell divisions for a prolonged period of time.

**Fig 5:**
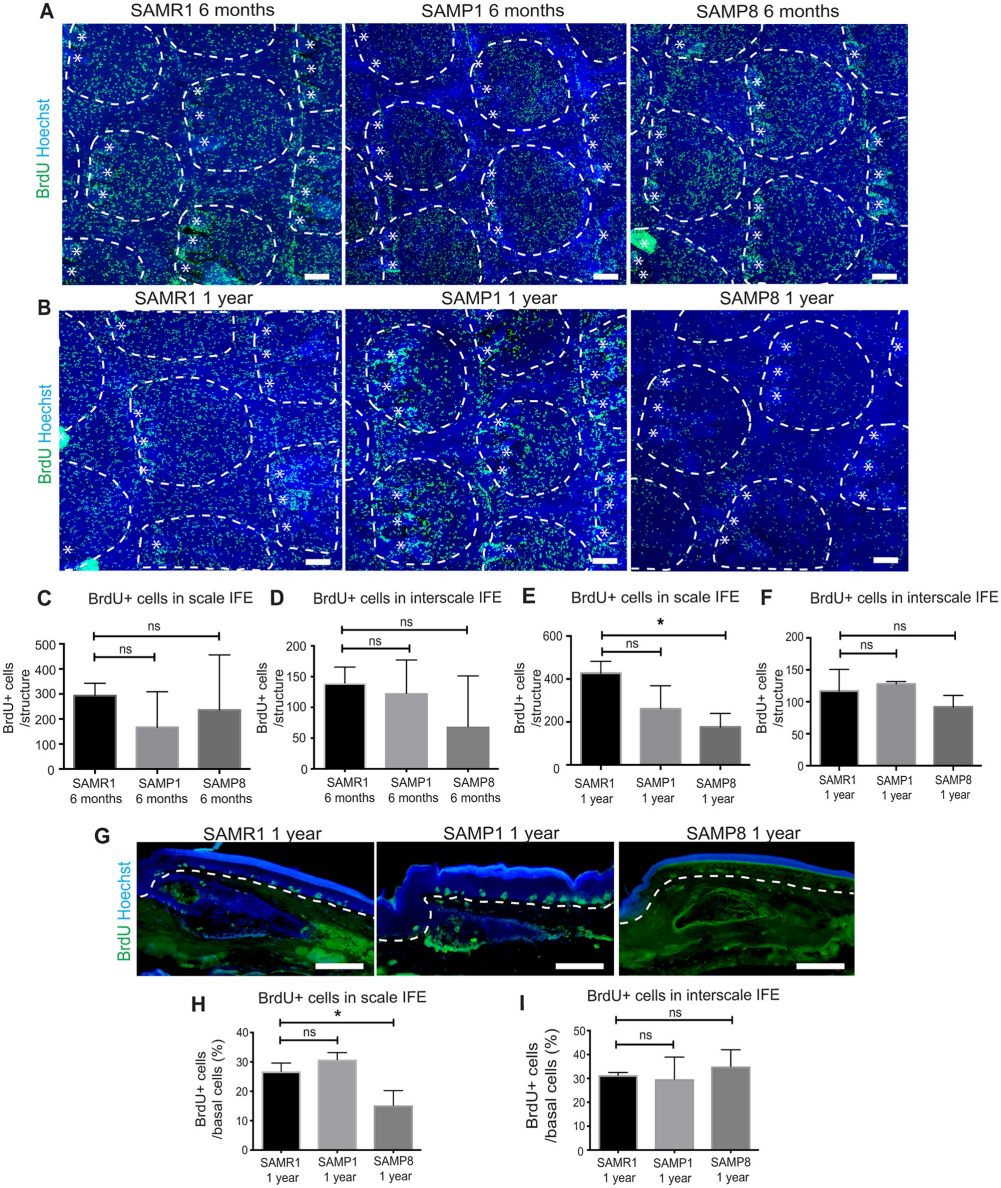
Decrease in scale proliferation in senescence prone SAMP8 mice. (**A, B**) Tail whole-mount staining of senescence-resistant SAMR1 and senescence-prone SAMP1 and SAMP8 mice at 6 months (A) and 1 year (B) with BrdU (proliferation marker, green) and Hoechst (blue). White dotted lines represent the boundary of scale and interscale. Asterisks represent hair follicles. Scale bars: 100 μm. (**C-F**) Quantification of BrdU-positive cells per scale or interscale units among SAM mice at 6 months (C, D) or 1 year (E, F) of age. The number of BrdU+ cells is scored from ≥8 interscale or scale IFE structures per mouse. *N* = 3. Error bars show S.E.M. One-way ANOVA. ns: not significant. (C) SAMR1 vs. SAMP1; *P* = 0.53. SAMR1 vs. SAMP8; *P* = 0.86. (D) SAMR1 vs. SAMP1; *P* = 0.91. SAMR1 vs. SAMP8; *P* = 0.30. (E) SAMR1 vs. SAMP1; *P* = 0.07. SAMR1 vs. SAMP8; *; *P* < 0.05. (F) SAMR1 vs. SAMP1; *P* = 0.77. SAMR1 vs. SAMP8; *P* = 0.35. (**G**) BrdU (proliferation marker, green) and Hoechst (blue) staining in the tail sections of SAMR, SAMP1 and SAMP8 at 1 year. (**H, I**) Proportion of BrdU+ cells per basal cells in the scale (H) or interscale IFE (I). The number of BrdU+ cells is scored from ≥100 basal cells per mouse. *N* = 3. One way ANOVA used. ns: not significant; (H) SAMR1 vs. SAMP1; *P* = 0.35. SAMR1 vs. SAMP8; *; *P* < 0.05. (I) SAMR1 vs. SAMP1; *P* = 0.31. SAMR1 vs. SAMP8; *P* = 0.26.

In SAMP1 and SAMP8 mice, the number of BrdU+ cells in the tail IFE at 6 month of age was lower than that in age-matched SAMR1 controls, with certain degree of variation between individuals (Fig 5A, 5C and 5D). In contrast, at 1 year of age, BrdU+ cells were unanimously reduced in the tail IFE of all the SAMP8 mice examined (Fig 5B, 5E–5I). Similarly to the wild-type old mice, SAMP8 mice showed a significant reduction of BrdU+ cells in the scale IFE, but not in the interscale IFE (Fig 5E, 5F, 5H and 5I). The staining with Ki67 antibody further supported the decreased proliferation of the IFE in SAMP8 mice at 1 year of age (S3 Fig). Taken together, the fast-dividing stem cell compartment showed a reduction in cell proliferation during aging and that this age-related phenotype was accelerated in the SAMP8 model.

### Age-associated decrease in the intensity of differentiation marker of slow-cycling stem cells

**Fig 6:**
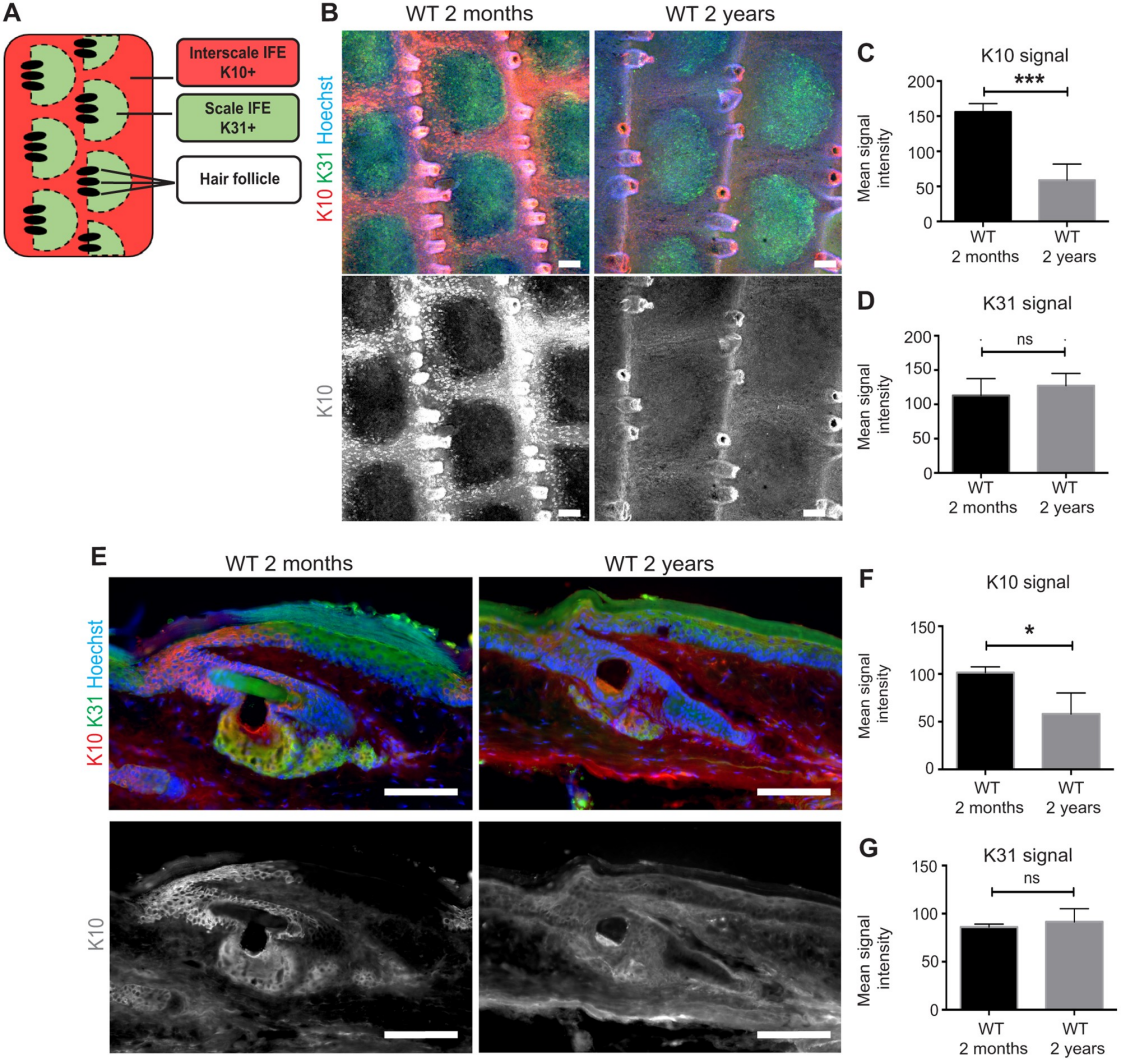
Age-dependent changes of the interscale differentiation marker. (**A**) Schematic view of the interscale and scale IFE differentiation in the tail whole-mount epidermis. (**B**) Young and old tail epidermis stained with Keratin (K) 10 (differentiation marker of interscale, red), K31 (differentiation marker of scale, green) and Hoechst (blue). The greyscale images show K10 staining. Scale bars: 100 μm. (**C, D**) Quantification of the mean signal intensity of K10 (C) and K31 (D) from young and old tail whole-mount epidermis. *N* = 4. Error bars show S.E.M. Student’s t-test. ***; *P* < 0.001 (C). ns: not significant; *P* = 0.38 (D). (**E**) Young and old tail epidermis sections stained with K10 (red), K31 (green) and Hoechst (blue). The greyscale images show the K10 staining. Scale bars: 100 μm. (**F, G**) Quantification of the mean signal intensity of K10 (F) and K31 (G). The intensity of immunostaining signals in skin section is measured and averaged from ≥50 individual cells per mouse. *N* = 3. Student’s t-test. *; *P* < 0.05 (F). ns: not significant; *P* = 0.54 (G).

To address whether differentiation of slow-cycling and fast-dividing stem cells were affected during aging, we performed tail whole-mount immuostaining with K10 and K31, to mark the orthokeratotic and parakeratotic differentiation, respectively (Fig 6A) [13]. In wild-types, the K10 intensity was significantly decreased in the interscale regions of old mice, whereas K31 staining pattern and intensity remained largely unchanged (Fig 6B–6D). To quantify the signal intensity from individual cells, we performed K10 and K31 staining in tail skin sections. Consistent with our observations on the whole-mount staining, the K10 signal intensity in sections was reduced with age, whereas K31 signal did not show any alterations in the staining pattern or intensity (Fig 6E–6G).

**Fig 7:**
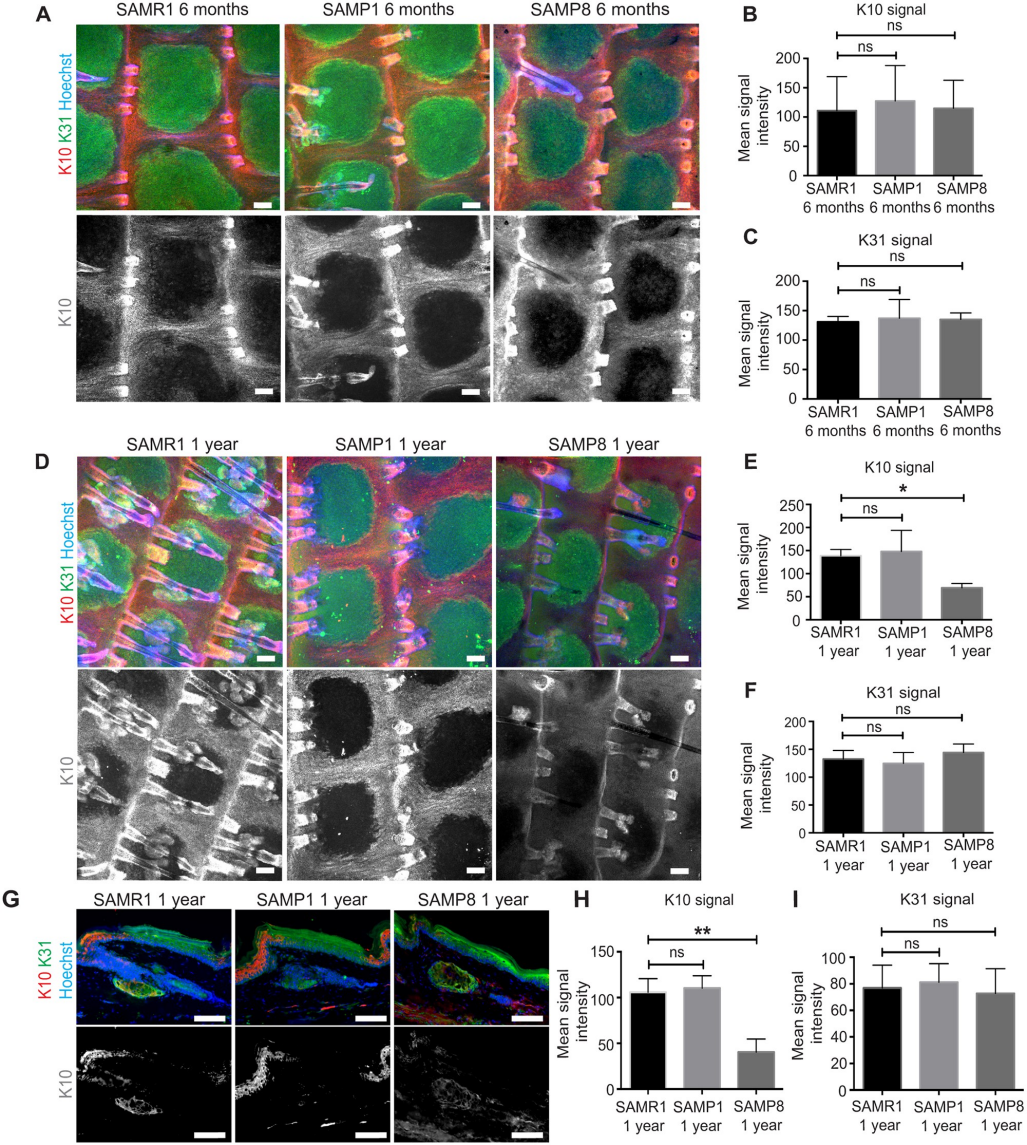
Decrease in the interscale differentiation marker in senescence prone SAMP8 mice. (**A**) Whole-mount tail epidermis stained with K10 (differentiation marker of interscale, red), K31 (differentiation marker of scale, green) and Hoechst (blue) of 6-month-old SAM mice. The grey scale images show K10 staining. Scale bars: 100 μm. (**B, C**) Quantification of the mean fluorescence intensity of K10 (B) and K31 (C). *N* = 3. One-way ANOVA. Error bars show S.E.M. ns: not significant; (B) SAMR1 vs. SAMP1; *P* = 0.91. SAMR1 vs. SAMP8; *P* = 0.99. (C) SAMR1 vs. SAMP1; *P* = 0.90. SAMR1 vs. SAMP8; *P* = 0.95. (**D**) Whole-mount staining of K10, K31 and Hoechst in 1-year-old SAM mice. (**E, F**) Quantification of the mean fluorescence intensity of K10 (E) and K31 (F). Scale bars: 100 μm. (E) SAMR1 vs. SAMP1; *P* = 0.89. SAMR1 vs. SAMP8; *; *P* < 0.05. (F) SAMR1 vs. SAMP1; *P* = 0.80. SAMR1 vs. SAMP8; *P* = 0.62. (**G**) Tail sections of SAMR, SAMP1 and SAMP8 mice stained with K10 (red), K31 (green) and Hoechst (blue) at 1 year of age. The greyscale images show the K10 staining. Scale bars: 100 μm. (**H, I**) Quantification of the mean signal intensity of K10 (H) and K31 (I). The intensity of immunostaining signals in skin section is measured and averaged from ≥50 individual cells per mouse. *N* = 3. One-way ANOVA. (H) SAMR1 vs. SAMP1; *P* = 0.90. SAMR1 vs. SAMP8; **; *P* < 0.01. (I) SAMR1 vs. SAMP1; *P* = 0.93. SAMR1 vs. SAMP8; *P* = 0.92.

Similar observations were obtained with senescence-accelerated SAMP8 mice at 1 year of age (Fig 7A–7I): K10 signal was significantly decreased in the interscale IFE compared to the age-matched SAMR1 controls (Fig 7D–7I), whereas SAMP1 mice did not show any obvious changes in the K10 expression either at 6 months or 1 year (Fig 7A–7I). Overall, the pattern of K31 staining remained unchanged in both SAMP8 and SAMP1 compared to age-matched SAMR1 controls (Fig 7C, 7F and 7I). Given that the loss of K10 is associated with impairments in epidermal desmosomal structures and the tissue integrity [45–48], our observation indicates that the differentiation program of slow-cycling stem cells may be misregulated in the aged IFE.

## Discussion

The mouse IFE is maintained by two distinct populations of epidermal stem cells that divide at a different rate [12]. Our current study focused on the age-dependent changes of slow-cycling and fast-dividing stem cells located in the interscale and scale IFE of tail skin, respectively. Considering that fast-dividing stem cells experience three times more divisions than slow-cycling stem cells over the entire life span [12], they may lose their stem cell property prematurely due to life-long, repeated cell divisions. Our observations revealed compartment-specific changes of epidermal stem cells during aging that were marked by distinct parameters. We found that fast-dividing stem cells showed a significant decrease in proliferation with age (Fig 4B and 4C), along with structural changes in the form of irregular micro-undulations and overall thinning of the epidermis (Fig 1B–1D). In contrast, the differentiation process is found to be more affected in the slow-cycling stem cell lineage (Fig 6B and 6C). These results suggest that distinct stem cell populations and their niches may react differently to aging. Further experiments are needed to address detailed cellular and molecular mechanisms of epidermal stem cell aging.

In the aged mouse IFE, we observed increased micro-undulations with the size of ~10 μm in the basal region (Fig 1B). The micro-undulations in old mice differ from the characteristic “rete ridge” structures present in the young human epidermis, which form much larger undulations with the size of 50–200 μm in depth/width [49]. We initially hypothesized that the age-associated increase of cell death in the basal layer could lead to the micro-undulations in the mouse IFE. However, our results point out that the apoptosis of basal cells does not change with age (Fig 3A–3H). Hence, we suppose that a decreased density of basal cells and/or irregular timing of delamination may contribute to the apparence of basal micro-undulations in the aged mouse skin.

In the current study, we compared histological, cellular and molecular differences between senescence-accelerated SAMP1 and SAMP8 with age-matched senescence-resistant SAMR1 as well as more senior wild-type C57BL/6, a commonly-used inbred strain of laboratory mouse. It has been suggested that the SAMP1 strain exhibited properties of photoaging in dorsal skin probably due to increased oxidative stress, which is commonly seen in the human skin with chronic UV exposure [29]. However, we did not observe an increase in proliferation or the thickness of the IFE in the tail skin of SAMP1 mice, as compared to age-matched SAMR1 controls (Fig 5, S3 Fig). This could be due to differences in age (1-year-old vs 1.5-year-old) or parts of skin (tail vs dorsal skin) used in our and their [29] studies. In contrast, SAMP8 mice at 1-year-old showed age-associated characteristics in tissue histology (Figs 1B and 2E), proliferation (Figs 4B and 5B) and differentiation (Figs 6B and 7D), which were comparable to 2-year-old mice of the C57BL/6 strain. Thus, we propose that the SAMP8 strain can potentially be a suitable model for understanding an aging process of epidermal stem cells and their niches.

Each strain of senescence-accelerated mice shows a strain-specific, aging-like phenotype and exome sequencing reveals the strain-specific mutations that could be responsible for these differences [50]. SAMP8 mice show an age-dependent decrease in the expression of genes that govern mitochondrial integrity and membrane potential, which could affect the fitness of mitochondria upon aging and result in an overall increase of oxidative stress across various organ systems [51–56]. Chronic treatments with growth hormone and melatonin have been shown to ameliorate the age-related phenotypes in the SAMP8 mice by targeting the oxidative status [57]. We presume that an oxidative stress may be the primary cause of premature aging of the SAMP8 skin, however we need further studies to address the occurrence and the level of oxidative stress in the SAMP8, as well as in wild-type old mice, with respect to the heterogeneious stem cell populations and their microenvironment.

## Supporting information

S1 Fig Basal marker expressions in the SAM mouse model. (**A**) Tail skin sections of senescence-resistant SAMR1 and senescence-prone SAMP1 and SAMP8 mice at 1 year of age are immunostained with α6 integrin (green), Collagen IV (red) and Hoechst (blue). The area within the yellow boxes are shown with higher magnification. Scale bars: 100 μm. The area within the yellow boxes are shown with higher magnification. Scale bars: 20 μm. Arrowheads indicate the micro-undulation. (**B**) Quantification of the mean signal intensity of α6 integrin. The intensity of immunostaining signals in skin section is measured and averaged from ≥50 individual cells per mouse. *N* = 3. Error bars show S.E.M. One-way ANOVA. ns: not significant; SAMR1 vs. SAMP1; *P* = 0.59. SAMR1 vs. SAMP8; *P* = 0.63.
(TIF)

S2 Fig Apoptotic cells in the SAM mouse model. (**A, B**) Tail whole-mount epidermal sheets of senescence-resistant SAMR1 and senescence-prone SAMP1 and SAMP8 mice at 6 months (A) and 1 year (B) of age are immunostained with cleaved caspase-3 (red), β4 integrin (green) and Hoechst (blue). White dotted lines represent the boundary of scale and interscale regions. Area within red dotted square is subjected for high magnification and shown on the right. Scale bars: 100 μm (left) or 20 μm (right).
(TIF)

S3 Fig Ki67+ proliferating cells in the SAM mouse model. (**A, B**) Tail whole-mount epidermal sheets of senescence-resistant SAMR1 and senescence-prone SAMP1 and SAMP8 mice at 6 months (A) and 1 year (B) of age are immunostained with Ki67 (proliferative marker, green) and Hoechst (blue). White dotted lines represent the boundary of scale and interscale. Asterisks represent hair follicles. Scale bars: 100 μm.
(TIF)

S1 Data Raw data for statistical analysis. (XLSX)
