# Supplementary figures and images for: Wild-type and SAMP8 mice show age-dependent changes in distinct stem cell compartments of the interfollicular epidermis
Source: PLoS One. 2019 May 15;14(5):e0215908. doi: 10.1371/journal.pone.0215908 (PMC6519801; doi:10.1371/journal.pone.0215908)

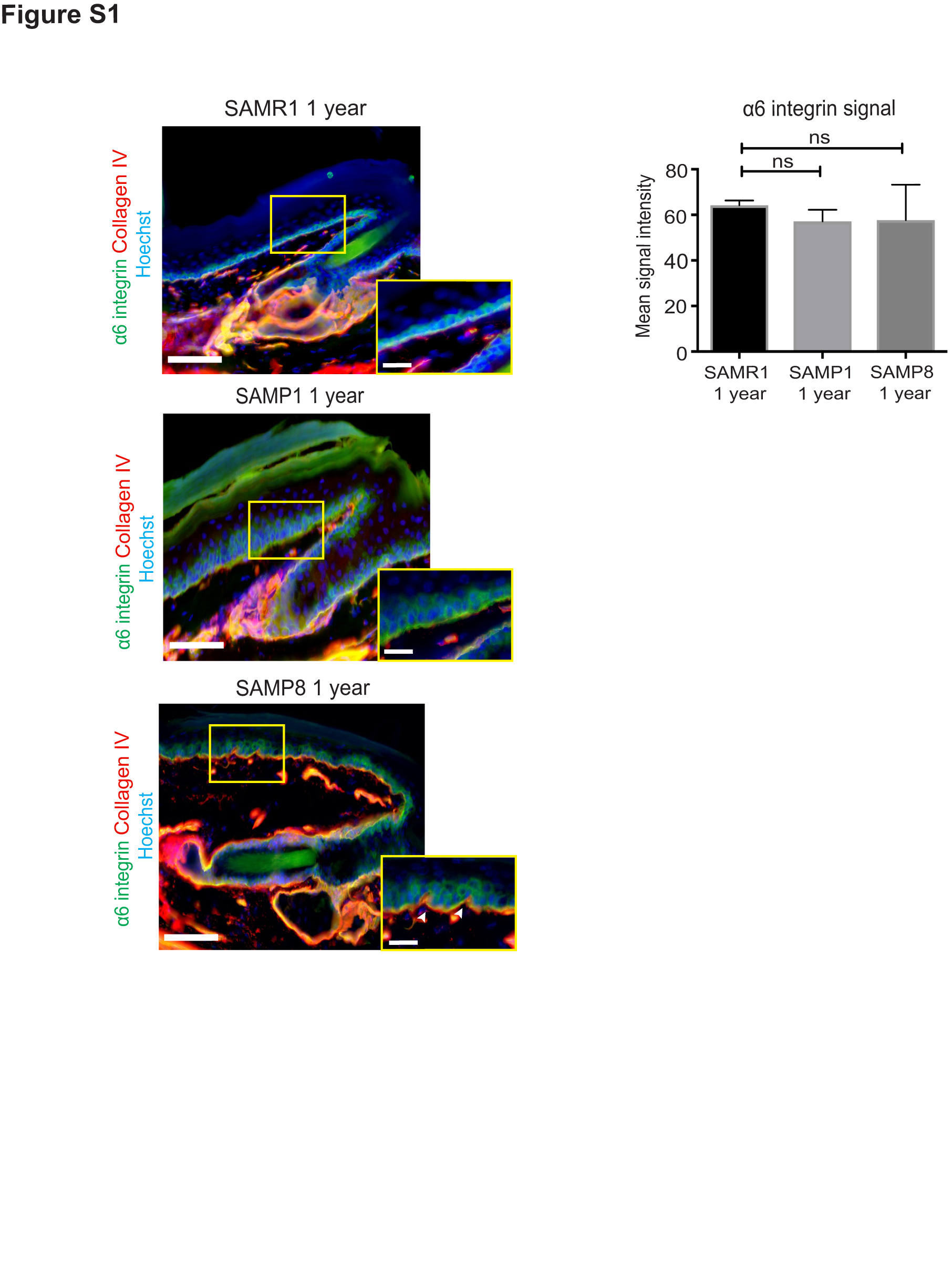

Supplement: S1 Fig — (A) Tail skin sections of senescence-resistant SAMR1 and senescence-prone SAMP1 and SAMP8 mice at 1 year of age are immunostained with α6 integrin (green), Collagen IV (red) and Hoechst (blue). The area within the yellow boxes are shown with higher magnification. Scale bars: 100 μm. The area within the yellow boxes are shown with higher magnification. Scale bars: 20 μm. Arrowheads indicate the micro-undulation. (B) Quantification of the mean signal intensity of α6 integrin. The intensity of immunostaining signals in skin section is measured and averaged from ≥50 individual cells per mouse. N = 3. Error bars show S.E.M. One-way ANOVA. ns: not significant; SAMR1 vs. SAMP1; P = 0.59. SAMR1 vs. SAMP8; P = 0.63. (TIF) [file pone.0215908.s001.tif]

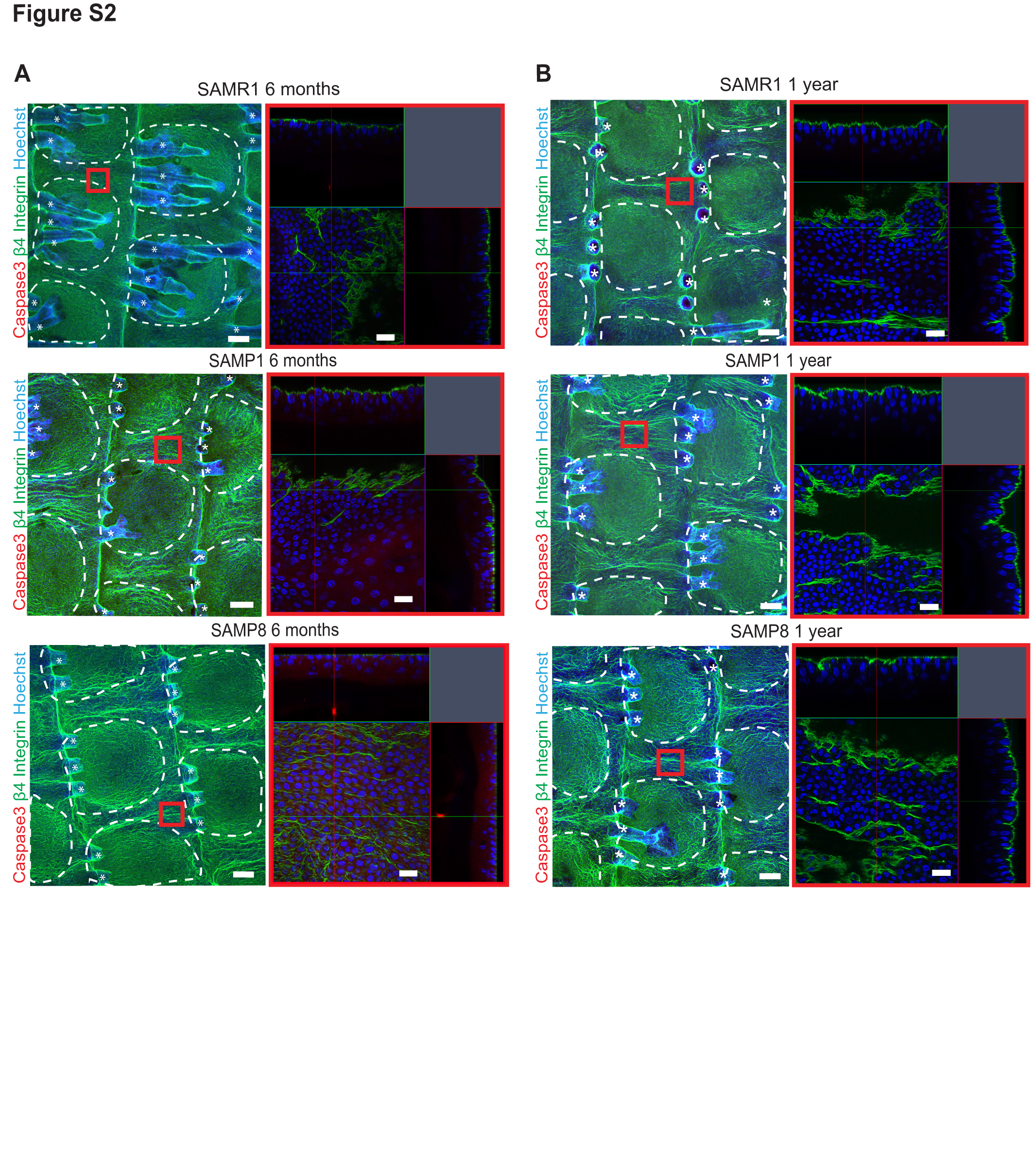

Supplement: S2 Fig — (A, B) Tail whole-mount epidermal sheets of senescence-resistant SAMR1 and senescence-prone SAMP1 and SAMP8 mice at 6 months (A) and 1 year (B) of age are immunostained with cleaved caspase-3 (red), β4 integrin (green) and Hoechst (blue). White dotted lines represent the boundary of scale and interscale regions. Area within red dotted square is subjected for high magnification and shown on the right. Scale bars: 100 μm (left) or 20 μm (right). (TIF) [file pone.0215908.s002.tif]

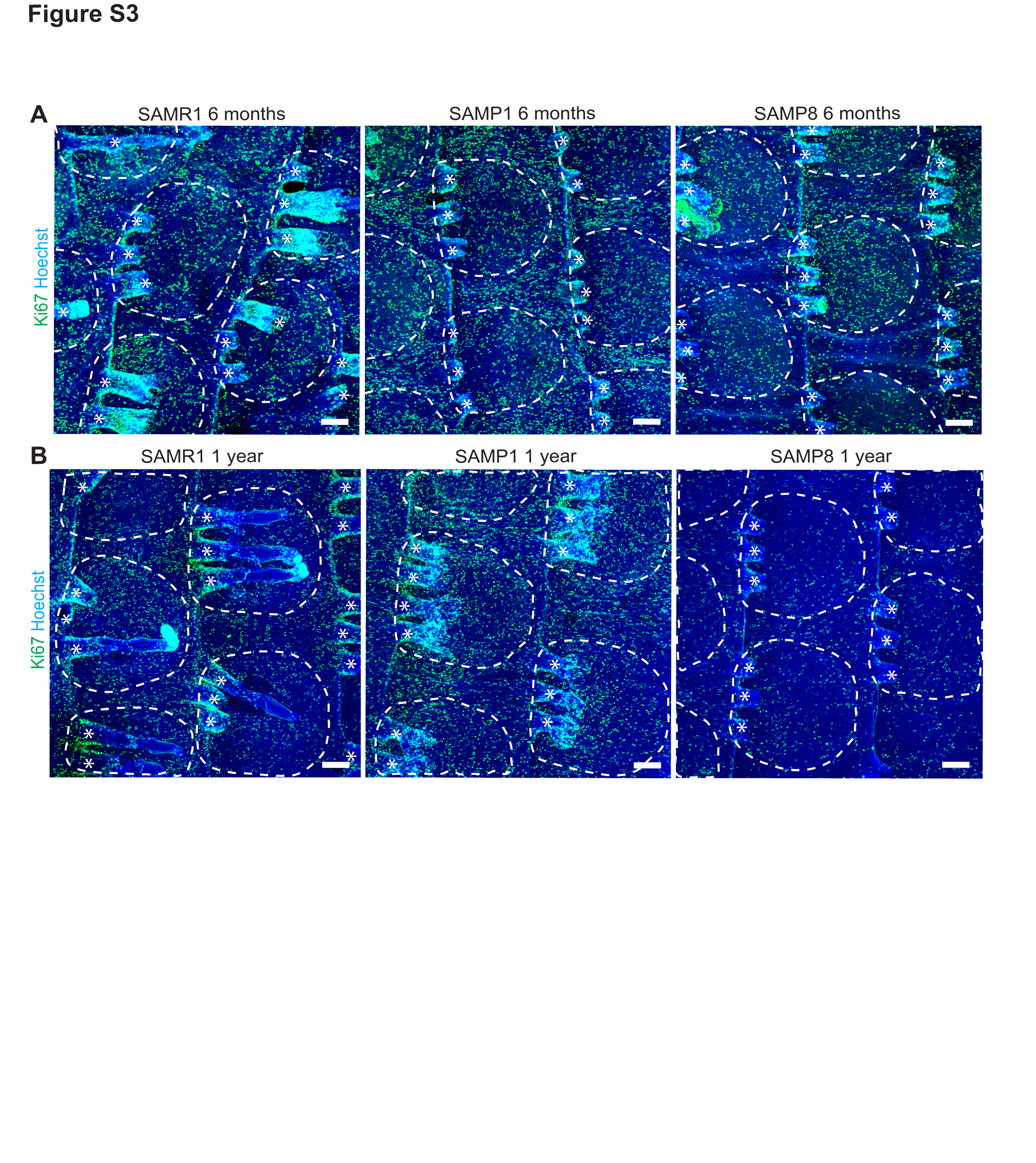

Supplement: S3 Fig — (A, B) Tail whole-mount epidermal sheets of senescence-resistant SAMR1 and senescence-prone SAMP1 and SAMP8 mice at 6 months (A) and 1 year (B) of age are immunostained with Ki67 (proliferative marker, green) and Hoechst (blue). White dotted lines represent the boundary of scale and interscale. Asterisks represent hair follicles. Scale bars: 100 μm. (TIF) [file pone.0215908.s003.tif]
